# Colour Doppler Ultrasonography in the Assessment of Intratesticular Lesions: Influence of Lesion Size and Vascular Pattern

**DOI:** 10.3390/cancers18050741

**Published:** 2026-02-25

**Authors:** Emily C. Bartlett, Dean Y. Huang, Marta Piorkowska, Maria E. Sellars, Jane L. Clarke, Seshadri Sriprasad, Gordon H. Muir, Daniel J. Quinlan, Paul S. Sidhu

**Affiliations:** 1 Department of Radiology, Royal Brompton Hospital, NHLI, Imperial College London, London SW3 6NP, UK; 2 Department of Clinical Radiology, King’s College Hospital, London SE5 9RS, UK; 3 Department of Imaging Sciences, School of Biomedical Engineering and Imaging Sciences, Faculty of Life Sciences and Medicine, King’s College London, London SE1 7EH, UK; 4 KHP Centre for Translational Medicine, King’s Health Partners, London SE5 9RS, UK; 5 Department of Radiology, University Hospital Lewisham, London SE13 6LH, UK; 6 Department of Urology, Darent Valley Hospital, Dartford DA2 8DA, UK; 7 Department of Urology, King’s College Hospital, London SE5 9RS, UK

**Keywords:** testicular tumour, intratesticular lesion, colour Doppler ultrasound, vascularity, intralesional blood flow, vascular pattern, germ cell tumour, orchiectomy, scrotal ultrasound

## Abstract

**Background/Objectives**: Conventional Colour Doppler Ultrasonography (CDUS) is widely used to assess vascularity in focal intratesticular lesions, yet the influence of lesion size on flow detection and the diagnostic utility of vascular distribution patterns remain unclear. We evaluated (i) whether lesion size affects CDUS detection of intralesional vascularity and (ii) whether vascular patterns associated with disruption of normal intratesticular vascular architecture are linked to diagnostic groupings. **Methods**: This retrospective single-centre study screened 12,189 testicular ultrasound examinations (1999–2009) and included histologically confirmed focal lesions with archived greyscale and CDUS images. To avoid within-patient clustering, one lesion per patient (the largest if multiple) was analysed (99 patients/lesions). Two blinded radiologists assessed vascularity (present/absent) and, for vascularised lesions, peripheral vascularity and intralesional patterns (criss-cross; disordered/haphazard). A derived composite “disrupted” pattern comprised criss-cross or disordered/haphazard flow. **Results**: Intralesional vascularity was present in 85/99 (85.9%) lesions. Vascularity was more common in neoplastic vs. non-neoplastic lesions (78/82 [95.1%] vs. 7/17 [58.8%]; *p* < 0.001) and in malignant vs. benign lesions (64/68 [94.1%] vs. 21/31 [67.7%]; *p* = 0.001). Lesion size was not associated with vascularity (the smallest vascularised lesion was 2 mm; logistic regression was non-significant). Among vascularised lesions, the composite disrupted pattern was more frequent in neoplastic vs. non-neoplastic (OR 11.67) and malignant vs. benign lesions (OR 5.90). Four of 14 avascular lesions were malignant/neoplastic. **Conclusions**: With optimised settings, CDUS vascularity detection did not appear size-limited and was strongly associated with neoplasia and malignancy, although avascularity did not exclude malignancy. A composite disrupted vascular pattern may be a practical, reproducible reporting descriptor warranting prospective validation.

## Simple Summary

Ultrasound is used to investigate testicular lumps, and more scanning means more small focal lesions are being found—many of which are not cancer. Clinicians often use Colour Doppler Ultrasound to look for blood flow within a lesion, but it is uncertain whether very small lesions seem to have no flow because of technical limits, and whether vessel patterns add diagnostic value. We reviewed 99 men with focal intratesticular lesions with final tissue diagnosis and re-analysed Doppler images for blood flow and for patterns suggesting disrupted normal vessels. Blood flow was far more common in tumours and cancers, and we detected flow in lesions as small as 2 mm, suggesting size alone is not the main barrier. However, some cancers had no detectable flow. A simple “disrupted flow” descriptor may help routine reporting and guide future studies.

## 1. Introduction

Ultrasonography (US) is the primary imaging modality for scrotal pathology because it is widely available, inexpensive and offers high spatial resolution [1]. Increased use of US has also challenged the historical assumption that all intratesticular lesions are malignant, with many focal lesions now recognised as benign or non-neoplastic [2,3]. This distinction is clinically important, particularly in younger men in whom appropriate selection for testis-sparing management may preserve fertility and endocrine function [4,5,6].

Despite the emergence of advanced techniques such as contrast-enhanced ultrasound and microvascular imaging, conventional colour Doppler ultrasonography (CDUS) remains the most commonly used method for vascular assessment in routine practice. The presence of intralesional vascularity on CDUS is generally considered a concerning feature, whereas benign and non-neoplastic entities are often presumed to be avascular [7]. However, the performance of CDUS in small lesions and the utility of vascular distribution patterns remain uncertain. In an early series, vascular detection was suggested to be size-dependent, with lesions <16 mm frequently appearing hypovascular [8]. Later experience suggests that intralesional flow may be demonstrable even in very small lesions when Doppler settings are optimised [9,10,11].

Interpretation of vascular patterns is further complicated by the normal testicular vascular architecture. After entering the scrotum, the testicular artery gives rise to capsular vessels and centripetal branches, producing a largely radial orientation of intratesticular vessels toward the mediastinum on colour Doppler imaging [12,13]. While disruption of this pattern might plausibly indicate neoplastic angiogenesis or infiltrative disease, the intrinsic vascular organisation of focal testicular lesions has been described inconsistently. Early reports emphasised an overall increased Doppler signal within malignant lesions without a reliable correlation between tumour cell type and vascular pattern [8]. Conversely, other series have proposed that malignancies may show disruption of normal vascular planes, including a characteristic “criss-cross” appearance [14], whereas some benign neoplasms such as Leydig cell tumours may demonstrate peripheral or mixed vascularity [15,16,17]. Reports on lymphoma have also suggested that preservation of normal vascular architecture may occur in a substantial proportion of cases [18], highlighting that “malignant” does not necessarily equate to a single stereotyped pattern. Collectively, these findings indicate that the relationship between vascular distribution and pathology is not yet conclusive, and that clinically pragmatic pattern descriptors may be required.

The aim of this study was to evaluate, in a series of histologically proven focal intratesticular lesions, (i) whether lesion size influences CDUS detection of intralesional vascularity, and (ii) whether specific vascular distribution patterns—particularly those suggestive of disruption of the normal radial/centripetal vascular organisation—are associated with diagnostic groupings.

## 2. Materials and Methods

### 2.1. Study Design and Patients

This was a retrospective, single-centre study performed in accordance with National Health Service (NHS) guidance. Institutional review board approval was waived for this retrospective review of existing clinical data and imaging, and the requirement for individual informed consent was waived.

**Figure 1:**
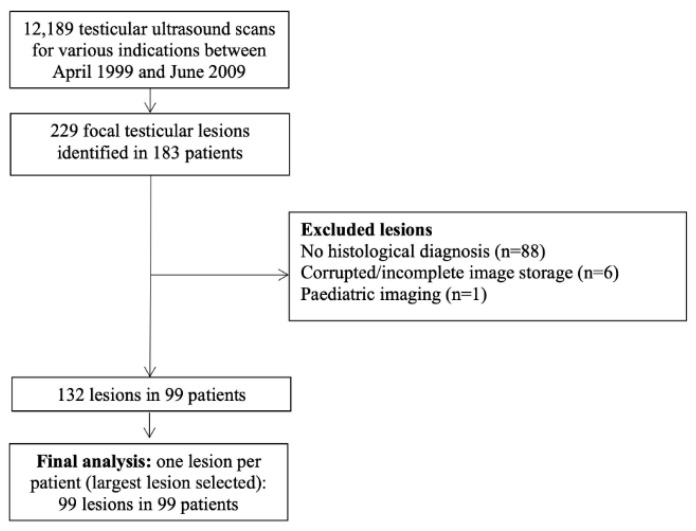
Flowchart of patients demonstrating reasons for exclusion. Lesions excluded for ‘lack of histological diagnosis’ were managed without surgery and therefore had no tissue reference standard.

A departmental database of 12,189 testicular ultrasound examinations performed between April 1999 and June 2009 was screened. This interval reflects the coverage of the departmental database used for case identification and coincided with a consistent acquisition environment (single operator/platform and standardised protocol), reducing technical heterogeneity for retrospective review. A total of 229 focal intratesticular lesions were identified in 183 patients. Inclusion criteria were: (1) a focal intratesticular lesion on ultrasound; (2) histopathological confirmation; and (3) availability of archived greyscale and colour Doppler ultrasound images in two orthogonal planes for retrospective review. Lesions were excluded for: lack of histological diagnosis (n = 88), corrupted or incomplete image storage (n = 6), or paediatric imaging (n = 1). Lack of histological diagnosis primarily reflects lesions managed without surgery (and therefore without a tissue reference standard), which were ineligible for inclusion by study design. After exclusions, 132 lesions in 99 patients remained (Figure 1). To avoid within-patient clustering, the primary analysis included one lesion per patient, defined as the largest lesion when multiple lesions were present. The final analysed cohort comprised 99 patients (99 lesions).

### 2.2. Ultrasound Examination

All examinations were performed by a single experienced operator (with >10 years’ experience in testicular ultrasound at the start of the study period) using a standardised departmental protocol. Ultrasound was performed using an Acuson Sequoia 512 system with a linear broadband transducer (15Lw; Acuson, Mountain View, CA, USA). Colour Doppler ultrasonography in this study refers to conventional colour-flow mapping (colour flow display) as implemented on the Acuson Sequoia system; no power Doppler or quantitative post-processing was used. Colour Doppler settings were optimised for testicular vascular assessment by adjusting pulse repetition frequency, gain and wall filter settings. Static images and cine loops in greyscale and colour Doppler modes were archived via PACS (GE Medical Systems, Barrington, IL, USA) and stored for retrospective analysis. Individual numeric Doppler parameter values (including gain) were not consistently stored or retrievable from the archived examinations.

### 2.3. Reference Standard and Diagnostic Grouping

Histopathology from surgical excision (orchidectomy or testis-sparing surgery, including procedures performed for diagnostic uncertainty) served as the reference standard. Lesions were grouped in two ways:

1. Neoplastic vs. non-neoplastic, where neoplastic lesions included germ cell tumours, lymphoma, leukaemia and Leydig/Sertoli cell tumours; non-neoplastic lesions included inflammatory, ischaemic/infarction-related, and developmental/benign non-neoplastic entities.
2. Benign vs. malignant, based on the final histological diagnosis.

### 2.4. Image Review and Definitions

Two radiologists (each with >4 years of experience in testicular ultrasound) independently reviewed imaging while blinded to histopathology. Recorded greyscale variables included maximum lesion diameter, echogenicity, border characteristics, and the presence of cystic change or calcification. Testicular microlithiasis and macrocalcifications were also recorded. Lesion volume was calculated as: length × width × height × 0.71.

Colour Doppler evaluation was performed in two steps:

(i) Vascularity (binary): Lesions were classified as vascularised (intralesional flow present) or avascular (no intralesional flow detected).
(ii) For vascularised lesions, peripheral vascularity was recorded as present/absent. Intralesional vascular organisation was classified into mutually exclusive patterns: criss-cross (crossing vessels within the lesion) or disordered/haphazard (disruption of the normal linear vascular pattern without definite vessel crossing). A derived composite ‘disrupted’ pattern was defined as the presence of either criss-cross or disordered/haphazard intralesional organisation.

Discrepancies between the two primary readers were resolved by consensus with an additional experienced observer (15 years of experience in testicular ultrasound). Inter-observer agreement analyses used the two independent (pre-consensus) ratings.

### 2.5. Statistical Analysis

Continuous variables are reported as mean ± standard deviation or median (interquartile range), as appropriate. Between-group comparisons of continuous variables were performed using the Mann–Whitney U test. Categorical variables were compared using Fisher’s exact test. Effect sizes for 2 × 2 comparisons are reported as odds ratios (ORs) with 95% confidence intervals (CIs). Primary vascularity analyses compared the presence of vascularity between (i) neoplastic vs. non-neoplastic lesions and (ii) benign vs. malignant lesions. Lesion size was evaluated in relation to vascularity using Mann–Whitney testing and univariable binary logistic regression, modelling vascularity as a function of maximum diameter and reporting OR per 1 mm increase. The maximum diameter is presented in mm. Vascular pattern analyses were restricted to vascularised lesions. Peripheral vascularity was analysed as a binary variable; criss-cross and disordered/haphazard intralesional patterns were analysed as mutually exclusive intralesional pattern subtypes and compared separately by outcome. Holm correction was applied across the three pattern tests (peripheral, criss-cross, disordered/haphazard) within each comparison (neoplastic vs. non-neoplastic; benign vs. malignant). For the seminoma vs. Leydig cell tumour subgroup comparison, Holm correction was similarly applied across the three individual patterns. Inter-observer agreement for peripheral vascularity, criss-cross pattern, disordered/haphazard pattern, and the derived composite disrupted pattern was assessed using Cohen’s kappa (κ) in the vascularised-lesion subset. All analyses were performed using SPSS (version 31). Two-sided *p*-values < 0.05 were considered statistically significant.

## 3. Results

### 3.1. Study Population

Ninety-nine examinations from 99 patients were included (mean age 38.2 years; median 35; range 19–79). As described in the Methods, the analyses were performed using one lesion per patient (the largest lesion when multiple were present). Lesions were left-sided in 53/99 (53.5%) and right-sided in 46/99 (46.5%). On final diagnosis, 31/99 (31.3%) lesions were benign, and 68/99 (68.7%) were malignant; patient age did not differ significantly between benign and malignant groups (*p* = 0.13). Circumscribed margins and hypoechogenicity were the most common greyscale appearances. Baseline cohort characteristics and greyscale ultrasound features stratified by benign versus malignant diagnosis are summarised in Table 1. Overall, malignant lesions comprised the majority of cases, with seminoma the most frequent malignant subtype; benign neoplasms were predominantly Leydig cell tumours, and the non-neoplastic group included inflammatory and ischaemic lesions (Table 2).

### 3.2. CDUS Vascularity

Colour Doppler demonstrated intralesional vascularity in 85/99 lesions (85.9%); 14/99 (14.1%) were avascular. Vascularity was more common in neoplastic than non-neoplastic lesions (78/82 [95.1%] vs. 7/17 [58.8%]; *p* < 0.001) and in malignant than benign lesions (64/68 [94.1%] vs. 21/31 [67.7%]; *p* = 0.001) (Table 3).

### 3.3. Lesion Maximum Diameter, Volume and Vascularity

Lesion size and volume are summarised in Table 4. There was no evidence of a difference in maximum diameter or volume between vascularised and avascular lesions (*p* = 0.888 and *p* = 0.692, respectively). The smallest vascularised lesion measured 2 mm (2 × 2 × 2 mm; volume 0.006 mL). On univariable logistic regression, maximum diameter (per 1 mm increase) was not associated with CDUS vascularity (Table 4).

### 3.4. Vascular Patterns in Vascularised Lesions (n = 85)

Inter-observer agreement was assessed using Cohen’s κ (n = 85). κ was 0.587 (SE 0.140; approximate 95% CI 0.31–0.86) for peripheral vascularity, 0.591 (SE 0.089; approximate 95% CI 0.42–0.77) for criss-cross, and 0.470 (SE 0.120; approximate 95% CI 0.24–0.71) for disordered/haphazard flow. Agreement for the derived composite “disrupted” pattern (criss-cross or disordered/haphazard) was higher (κ = 0.950; SE 0.050; approximate 95% CI 0.85–1.00) (Table S2).

**Figure 2:**
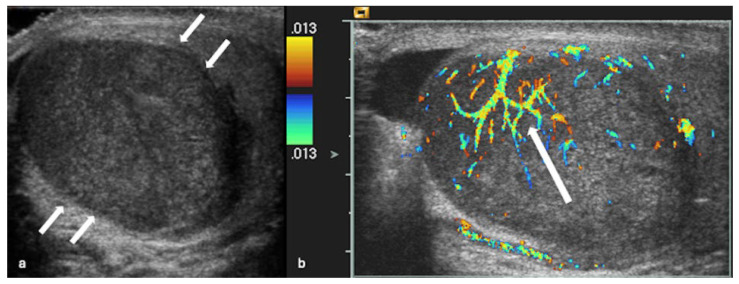
Representative colour Doppler appearances of seminoma. Transverse and longitudinal images of a seminoma in the left testis: (**a**) grey-scale ultrasound shows a well-circumscribed, hypoechoic focal intratesticular lesion (short arrows); (**b**) colour Doppler ultrasound demonstrates a criss-cross intralesional vascular pattern (long arrow).

**Figure 3:**
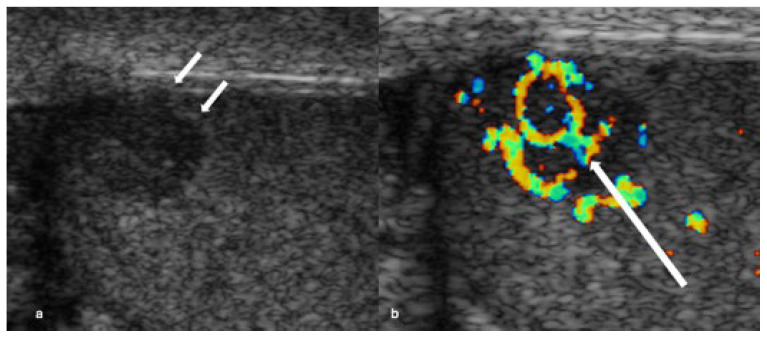
Benign Leydig cell tumour with a criss-cross intralesional vascular pattern. Longitudinal images of a benign Leydig cell tumour in the left testis: (**a**) grey-scale ultrasound shows a well-circumscribed hypoechoic lesion (8.0 × 5.0 × 5.0 mm; short arrows); (**b**) colour Doppler ultrasound demonstrates a criss-cross intralesional vascular pattern (long arrow), which can mimic appearances seen in malignant neoplastic lesions.

**Figure 4:**
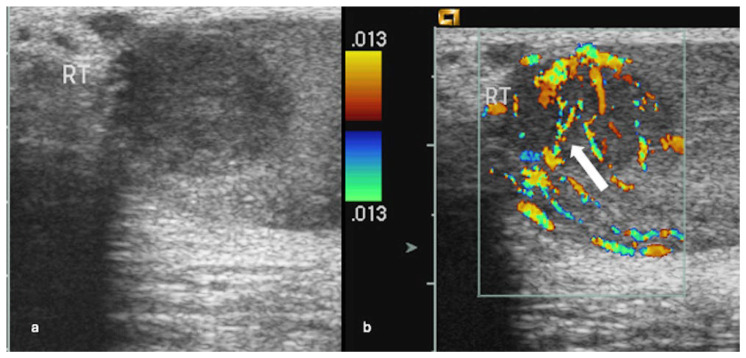
Disordered/haphazard intralesional vascular pattern in a mixed germ cell tumour. (**a**) Grey-scale ultrasound shows a well-circumscribed hypoechoic lesion in the right testis: (**b**) colour Doppler ultrasound demonstrates non-crossing disordered/haphazard intralesional vascularity (arrow).

**Figure 5:**
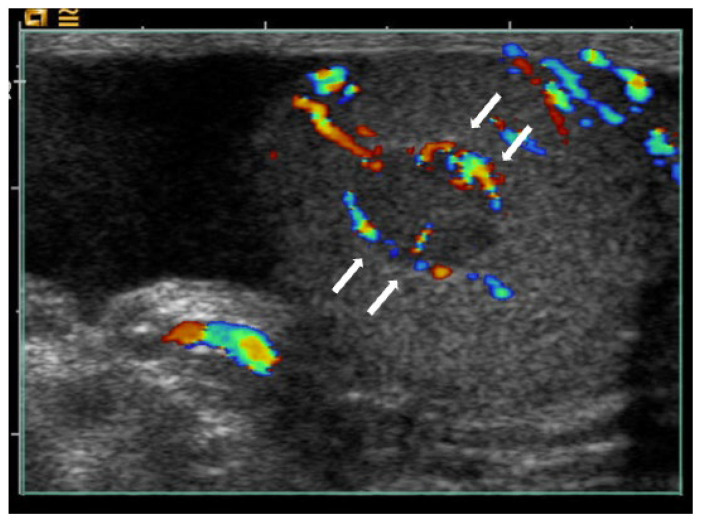
Peripheral vascularity in a small benign Leydig cell tumour. Longitudinal colour Doppler ultrasound images of the left testis show a well-circumscribed hypoechoic intratesticular lesion measuring 5 mm, demonstrating peripheral vascularity (short arrows).

Peripheral vascularity was recorded independently (non-exclusive). Criss-cross and disordered/haphazard intralesional patterns were mutually exclusive. Among vascularised lesions, criss-cross was present in 62/85 (72.9%) (Figure 2 and Figure 3), disordered/haphazard flow in 10/85 (11.8%) (Figure 4), and neither intralesional disrupted subtype in 13/85 (15.3%). Peripheral vascularity was present in 9/85 (10.6%) (Figure 5). Unadjusted and Holm-adjusted *p* values for the individual component-pattern tests are provided in Table S1.

#### 3.4.1. Neoplastic vs. Non-Neoplastic

Peripheral vascularity was observed in 9/78 (11.5%) neoplastic lesions and 0/7 (0%) non-neoplastic lesions (*p* = 1.000). Criss-cross was more frequent in neoplastic than non-neoplastic lesions (59/78 [75.6%] vs. 3/7 [42.9%]) but did not reach statistical significance (*p* = 0.082), and disordered/haphazard flow occurred in 10/78 (12.8%) versus 0/7 (0%) (*p* = 0.592). No individual component pattern remained statistically significant after adjustment (Table S1).

#### 3.4.2. Benign vs. Malignant

Peripheral vascularity was present in 3/21 (14.3%) benign versus 6/64 (9.4%) malignant lesions (*p* = 0.683). Criss-cross was present in 13/21 (61.9%) versus 49/64 (76.6%) (*p* = 0.257), and disordered/haphazard flow in 1/21 (4.8%) versus 9/64 (14.1%) (*p* = 0.439). No individual component pattern remained statistically significant after adjustment (Table S1).

#### 3.4.3. Seminoma Versus Leydig Cell Tumour

Criss-cross was nominally more frequent in seminoma than in Leydig cell tumours (33/34 [97.1%] vs. 10/14 [71.4%]; *p* = 0.021), but this did not remain significant after Holm correction (Holm-adjusted *p* = 0.063). Peripheral vascularity did not differ between groups (Holm-adjusted *p* = 0.400), and disordered/haphazard flow was uncommon (0/34 [0%] vs. 1/14 [7.1%]; *p* = 0.292; Holm-adjusted *p* = 0.584) (Table S3).

#### 3.4.4. Derived Composite “Disrupted” Pattern (Criss-Cross or Disordered/Haphazard)

The disrupted pattern was more frequent in neoplastic than non-neoplastic lesions (70/78 [89.7%] vs. 3/7 [42.9%]; *p* = 0.007; OR 11.67, 95% CI 2.21–61.73) and in malignant than benign lesions (59/64 [92.2%] vs. 14/21 [66.7%]; *p* = 0.008; OR 5.90, 95% CI 1.63–21.37) (Table 5). In the seminoma versus Leydig comparison, the disrupted pattern was present in 34/34 (100%) seminomas and 11/14 (78.6%) Leydig cell tumours (*p* = 0.021) (Table S3).

### 3.5. Avascular Lesions (n = 14)

**Figure 6:**
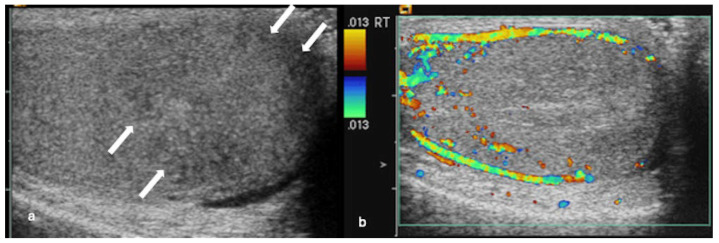
Segmental infarction. Longitudinal images of the left testis: (**a**) grey-scale ultrasound shows an ill-defined hypoechoic intratesticular lesion (arrows); (**b**) colour Doppler ultrasound demonstrates no detectable intralesional vascularity.

**Figure 7:**
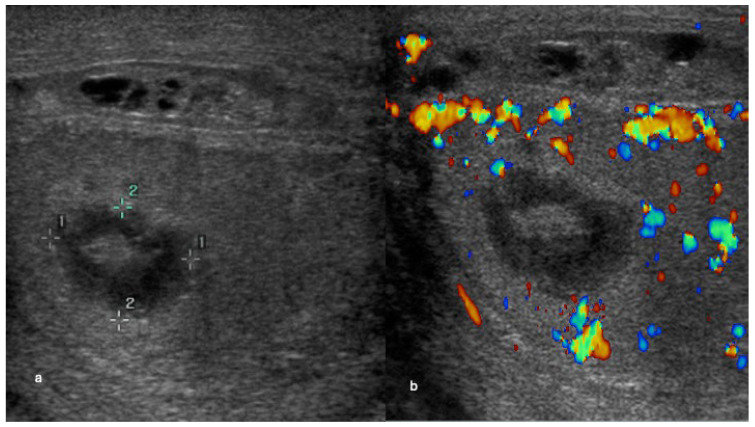
Avascular malignant testicular lesion (acute myeloid leukaemia involvement). Longitudinal images of the left testis: (**a**) grey-scale ultrasound shows a small focal intratesticular lesion (15 × 10 × 10 mm; callipers); (**b**) colour Doppler ultrasound demonstrates no detectable intralesional vascularity.

Among 14 avascular lesions, 4/14 were malignant/neoplastic, and 10/14 were non-neoplastic. A representative example of a non-neoplastic lesion (segmental infarction) is shown in Figure 6. Malignant diagnoses comprised a burnt-out tumour, a mixed germ cell tumour (seminoma 70%, yolk sac tumour 20%, embryonal carcinoma 10%), haematological malignancy involvement (AML/myelodysplasia), and metastatic adenocarcinoma (bowel primary) (each n = 1). Non-neoplastic diagnoses were most commonly segmental infarction (n = 3). Final diagnoses of avascular lesions are summarised in Table S4. A representative example of an avascular malignant lesion (testicular involvement by acute myeloid leukaemia) is shown in Figure 7.

## 4. Discussion

In this retrospective cohort of histologically confirmed focal intratesticular lesions, we evaluated whether lesion size influences colour Doppler ultrasound (CDUS) detection of vascularity and whether vascular distribution patterns help characterise underlying pathology. The principal findings were threefold. First, intralesional vascularity on CDUS was associated with both neoplasia and malignancy, being more frequent in neoplastic than non-neoplastic lesions and in malignant than benign lesions. Second, lesion size was not related to vascularity detection in this dataset. The smallest vascularised lesion measured 2 mm, and neither group comparison nor logistic regression supported a size-dependent reduction in detectable flow. Third, while individual pattern components (peripheral, criss-cross, disordered/haphazard) did not remain significant after multiplicity correction, a derived composite “disrupted” pattern (criss-cross or disordered/haphazard) was more frequent in malignant than benign lesions and in neoplastic than non-neoplastic lesions within the vascularised subset, suggesting potential value as a pragmatic summary descriptor of abnormal intralesional vascular organisation.

Our findings support the clinical premise that intralesional vascularity on conventional CDUS is associated with malignant and neoplastic pathology in focal testicular lesions [1,8,19]. In contrast to prior reports suggesting reduced Doppler conspicuity in very small lesions [8], we did not observe evidence that small lesion size alone limits vascular detection with optimised CDUS settings. Maximum diameter was not associated with vascularity on univariable logistic regression (per 1 mm increase). The smallest lesion measured 2 mm, showing that intralesional flow can be detected even at this size with optimised CDUS. This finding suggests that the absence of Doppler flow in a small lesion should not be assumed to represent a technical limitation in isolation. However, vascularity was not specific for malignancy: benign Leydig cell tumours were also frequently vascular, consistent with prior observations, including CEUS studies [20,21].

The composition of avascular lesions in our cohort is clinically relevant. Although avascularity was more common in non-neoplastic disease overall, a minority of avascular lesions were malignant/neoplastic, underscoring that the absence of Doppler flow does not exclude malignancy. Biologically, Doppler negativity in malignant lesions may reflect necrosis or haemorrhage, infarction, or low-flow/infiltrative disease below the detection threshold of colour Doppler, including in the context of a burnt-out tumour [22]. These findings support a cautious interpretation of Doppler negativity [7], particularly in lesions of raised clinical concern.

The current study was motivated by conflicting reports regarding whether intralesional vascular patterns—particularly those implying disruption of normal radial centripetal vascular architecture—could predict malignancy or specific histologies [14,16,17,18,23]. In the present cohort, peripheral vascularity was recorded independently, whereas criss-cross and disordered/haphazard intralesional patterns were classified as mutually exclusive subtypes (the latter defined by disruption without definite vessel crossing). When analysed individually, none of the three patterns remained significant (after Holm correction) in either the neoplastic vs. non-neoplastic or benign vs. malignant comparisons. This suggests that, taken separately, peripheral, criss-cross and disordered/haphazard appearances may be insufficiently specific to function as standalone discriminators.

The derived composite ‘disrupted’ pattern (criss-cross or disordered/haphazard) showed a consistent association with neoplasia and malignancy within vascularised lesions. This construct is anatomically plausible because both component patterns represent departures from the expected intratesticular vascular planes. Inter-observer agreement for the individual component patterns was moderate-to-substantial, whereas agreement for the derived composite was higher. This likely reflects that readers may differ when distinguishing criss-cross from disordered/haphazard appearances, yet can more consistently agree on the presence of any disrupted vascular organisation. These findings suggest that the composite descriptor may offer a pragmatic summary measure that may be more robust than individual component patterns alone. This analysis should be considered exploratory and warrants prospective validation.

A clinically important differential diagnosis for vascularised focal lesions is seminoma versus Leydig cell tumours, particularly in men being considered for testis-sparing management [3,24,25,26]. In our cohort, the criss-cross pattern was nominally more frequent in seminoma than in Leydig cell tumours, but this difference did not remain significant after Holm correction. This finding is suggestive but not definitive and is consistent with uncertainty in the published literature, supporting caution when making histology-specific inferences based on ultrasound appearances alone [10,20,27,28,29,30]. The criss-cross pattern on CDUS may contribute to the overall imaging impression when interpreted alongside other ultrasound parameters (i.e., a multiparametric ultrasound approach) [27,28,31], but should not be used in isolation to dictate management.

### 4.1. Clinical Implications

These results support a practical approach to CDUS interpretation in focal intratesticular lesions. Intralesional vascularity increases the likelihood of neoplasia and malignancy, but it is not specific, given that some benign lesions are vascularised and some malignant/neoplastic lesions are avascular. Lesion size alone should not be assumed to limit vascular detection when CDUS is optimised. If flow is absent, alternative biological explanations (e.g., infarction, fibrosis, necrosis) should be considered rather than attributing the finding to technical limitations. Finally, within vascularised lesions, reporting a composite “disrupted” vascular pattern may offer a reproducible way to communicate abnormal vascular organisation. Contrast-enhanced ultrasound (CEUS) may improve the detection of microvascular flow, but requires intravenous contrast and operator expertise, which may not be universally available [32]. Future studies could evaluate whether advanced microvascular Doppler techniques add incremental value over optimised CDUS for small lesions and lesions with equivocal flow.

### 4.2. Limitations

This study has several limitations. This was a retrospective, single-centre study conducted in an earlier era of ultrasound hardware; although colour Doppler settings were optimised, generalisability to other equipment and contemporary Doppler implementations may be limited. Accordingly, external validity to contemporary multi-platform practice requires prospective validation. Patients were recruited over a 10-year period, and CDUS acquisition parameters were not standardised or fully retrievable across the study period. This includes colour gain and related Doppler settings (e.g., PRF and wall filter), which can influence colour conspicuity and vascularity categorisation. Although imaging was performed by a single experienced operator who optimised settings at the time of scanning, some non-protocol variability is inevitable in a retrospective dataset. Archived CDUS remains susceptible to motion artefact, with potential non-differential misclassification of vascularity. Inclusion of histologically confirmed lesions introduces verification and spectrum bias towards surgically managed cases, potentially inflating the prevalence of malignancy and limiting generalisability to incidentally detected small lesions managed with surveillance. This is particularly relevant given the number of lesions excluded for lack of histology (n = 88). Non-neoplastic vascularised lesions were few (n = 7), limiting precision and reducing power for pattern-based discrimination in that subgroup. Selecting the largest lesion in patients with multiple lesions likely biases summaries towards more conspicuous lesions and does not capture within-testis heterogeneity in vascularity. Conventional colour Doppler may fail to display very low-level or slow flow; therefore, ‘no detectable flow’ on CDUS should not be interpreted as the absence of perfusion. Finally, the composite “disrupted” endpoint, while anatomically motivated and derived from coded components, remains an analytic construct; prospective validation with prespecified hypotheses and standardised pattern definitions would strengthen inference.

## 5. Conclusions

In histologically confirmed focal testicular lesions, intralesional vascularity on conventional CDUS was strongly associated with neoplastic and malignant pathology, and vascular detection did not appear to be constrained by lesion size. Within vascularised lesions, a composite “disrupted” vascular pattern was reproducible and more frequent in neoplastic and malignant lesions, supporting its consideration as a practical reporting descriptor, while individual pattern components showed limited standalone discriminatory value.

## Figures and Tables

**Table 1:** Patient and lesion characteristics and greyscale ultrasound features stratified by final diagnosis (n = 99).

Characteristic	Overall (n = 99)	Benign (n = 31)	Malignant (n = 68)
Age, years (mean; median)	38.25 ± 12.54; 35 (19–79)	40.00 ± 11.94; 38 (19–74)	37.46 ± 12.82; 35 (20–79)
Side (lesion laterality)			
Left	53 (53.5)	15 (48.4)	38 (55.9)
Right	46 (46.5)	16 (51.6)	30 (44.1)
Greyscale ultrasound features			
Margins			
Circumscribed/well-defined	79 (79.8)	21 (67.7)	58 (85.3)
Other/poorly defined	20 (20.2)	10 (32.3)	10 (14.7)
Echogenicity			
Hypoechoic	63 (63.6)	23 (74.2)	40 (58.8)
Other	36 (36.4)	8 (25.8)	28 (41.2)
Marked heterogeneity (present)	33 (33.3)	6 (19.4)	27 (39.7)
Microlithiasis (present)	18 (18.2)	3 (9.7)	15 (22.1)
Macrocalcification (present)	6 (6.1)	0 (0.0)	6 (8.8)

Data are n (%) unless otherwise stated. Percentages are column percentages. Age is reported as mean ± SD and median (range).

**Table 2:** Histological diagnosis distribution of focal intratesticular lesions (n = 99).

Group	Histology	n	% of Total (n = 99)	% Within Group
Malignant neoplastic	Classical seminoma	34	34.3	50
	Mixed germ cell tumour	12	12.1	17.6
	Malignant teratoma	8	8.1	11.8
	Acute myeloid leukaemia (AML)	4	4	5.9
	Embryonal carcinoma	2	2	2.9
	Lymphoma	2	2	2.9
	Rhabdomyosarcoma	1	1	1.5
	Metastasis—prostate	1	1	1.5
	Combined germ cell tumour (seminoma 70%, yolk sac 20%, embryonal 10%)	1	1	1.5
	AML with myelodysplasia	1	1	1.5
	Metastasis—adenocarcinoma from the bowel	1	1	1.5
	Burnt-out tumour	1	1	1.5
	Subtotal malignant neoplastic	68	68.7	100
Benign neoplastic	Leydig cell tumour with low malignant potential	14	14.1	100
	Subtotal benign neoplastic	14	14.1	100
Non-neoplastic	Acute segmental infarction	4	4	23.5
	Orchitis	3	3	17.6
	Fibrous scarring	2	2	11.8
	Venous infarction and chronic ischaemia	1	1	5.9
	Complete infarction	1	1	5.9
	Atrophy and pyocele	1	1	5.9
	Abscess	1	1	5.9
	Simple tunica cyst	1	1	5.9
	Splenic heterotopia	1	1	5.9
	No pathological abnormality identified (orchiectomy)	2	2	11.8
	Subtotal non-neoplastic	17	17.2	100
Total	Total	99	100	

Data are n (% of total, n = 99); within-group percentages are provided for malignant (n = 68), benign neoplastic (n = 14) and non-neoplastic (n = 17) categories.

**Table 3:** Association of intralesional CDUS vascularity with histological outcome (n = 99).

Comparison/Group	Avascular (No Flow)	Vascularised (Flow Present)	Fisher’s Exact *p*	Odds Ratio (95% CI)
Neoplastic status				
Non-neoplastic (n = 17)	10 (58.8%)	7 (41.2%)		
Neoplastic (n = 82)	4 (4.9%)	78 (95.1%)	<0.001	27.86 (6.91–112.26)
Malignancy status				
Benign (n = 31)	10 (32.3%)	21 (67.7%)		
Malignant (n = 68)	4 (5.9%)	64 (94.1%)	0.001	7.62 (2.16–26.86)

Data are n (%), with percentages calculated within rows. *p*-values are from Fisher’s exact test (two-sided). ORs are odds ratios for vascularity present (vascularised vs. avascular) comparing neoplastic vs. non-neoplastic and malignant vs. benign, with 95% confidence intervals.

**Table 4:** Lesion maximum diameter by vascularity status and univariable logistic regression for vascularity (n = 99).

	Avascular (n = 14)	Vascularised (n = 85)	*p* Value
Lesion size summary by vascularity status			
Maximum diameter (mm)	Median 18.5 [11.0–35.5]; range 6–54 Mean 23.29 ± 15.81	Median 21.0 [10.5–36.0]; range 2–100 Mean 26.31 ± 21.00	0.888 *
Univariable logistic regression (outcome: vascularised vs. avascular)			
Predictor (per unit)	OR	95% CI	*p* value
Maximum diameter (per 1 mm)	1.008	0.978–1.040	0.605

Lesion size is reported as maximum diameter (mm). Group summaries are median [IQR], range and mean ± SD. * Mann–Whitney U test (two-sided). Univariable logistic regression modelled vascularity (vascularised vs. avascular) as the outcome; the predictor was maximum diameter per 1 mm increase, reported as OR (95% CI) with Wald *p* value.

**Table 5:** Peripheral and composite “disrupted” colour Doppler vascular patterns in vascularised focal intratesticular lesions and their association with neoplasia and malignancy (n = 85).

Pattern (Present)	Group 1, n/N (%)	Group 2, n/N (%)	Fisher’s Exact *p*	OR (95% CI)
Panel A: Neoplastic vs. non-neoplastic (vascularised lesions only)				
Peripheral vascularity	Neoplastic (n = 78): 9/78 (11.5)	Non-neoplastic (n = 7): 0/7 (0.0)	1.000	2.05 (0.11–38.85) †
Composite “disrupted” pattern	Neoplastic (n = 78): 70/78 (89.7)	Non-neoplastic (n = 7): 3/7 (42.9)	0.007	11.67 (2.21–61.73)
Panel B: Benign vs. malignant (vascularised lesions only)				
Peripheral vascularity	Benign (n = 21): 3/21 (14.3)	Malignant (n = 64): 6/64 (9.4)	0.683	0.62 (0.14–2.74)
Composite “disrupted” pattern	Benign (n = 21): 14/21 (66.7)	Malignant (n = 64): 59/64 (92.2)	0.008	5.90 (1.63–21.37)

Data are restricted to vascularised lesions (intralesional flow present; n = 85). Values are n/N (%) within each diagnostic group. Peripheral vascularity was recorded as present/absent. The derived composite “disrupted” pattern was defined as criss-cross or disordered/haphazard intralesional organisation (component patterns mutually exclusive). Two-sided Fisher’s exact test was used for *p*-values. ORs (95% CIs) compare the odds of neoplasia (neoplastic vs. non-neoplastic) or malignancy (malignant vs. benign) when the pattern is present vs. absent. † For Panel A peripheral vascularity, a 0.5 continuity correction was applied to estimate OR and CI due to a zero cell; Fisher’s *p* value is unchanged.

## Data Availability

The data presented in this study are not publicly available due to patient confidentiality and institutional/NHS data governance restrictions.

## Supplementary Materials

The following supporting information can be downloaded at: https://www.mdpi.com/article/10.3390/cancers18050741/s1, Table S1. Individual colour Doppler vascular pattern components in vascularised focal intratesticular lesions with Holm multiplicity control (n = 85). Table S2. Inter-observer agreement for colour Doppler vascular pattern assessment in vascularised focal intratesticular lesions (n = 85). Table S3. Seminoma versus Leydig cell tumour (vascularised subset): colour Doppler vascular pattern component comparisons with Holm multiplicity control. Table S4. Histological diagnoses of avascular focal intratesticular lesions on colour Doppler ultrasonography (n = 14).
