# Supplementary material for: Colour Doppler Ultrasonography in the Assessment of Intratesticular Lesions: Influence of Lesion Size and Vascular Pattern
Source: Cancers (Basel). 2026 Feb 25;18(5):741. doi: 10.3390/cancers18050741 (PMC12984502; doi:10.3390/cancers18050741)
Supplement: Supplementary file 1 [file cancers-18-00741-s001.zip › TableS4.pdf]

Table S4. Histological diagnoses of avascular focal intratesticular lesions on colour Doppler ultrasonography (n = 14).

**Diagnosis**	n
Acute segmental infarction	3
Venous infarction and chronic ischaemia	1
Complete infarction	1
Atrophy and pyocele	1
Abscess	1
Simple tunica cyst	1
Fibrous scarring	1
No pathological abnormality identified (orchiectomy)	1
Burnt-out tumour	1
Combined germ cell tumour (seminoma 70%, yolk sac tumour 20%, embryonal carcinoma 10%)	1
AML / myelodysplasia	1
Adenocarcinoma from bowel (metastatic)	1
**Subtotal: malignant/neoplastic**	4
**Subtotal: non-neoplastic**	10

Avascular lesions were defined as no detectable intralesional colour Doppler flow on archived images. Values are counts of final diagnoses within the avascular subset (n = 14). Subtotals indicate malignant/neoplastic versus non-neoplastic diagnoses based on the final histology.
