# Supplementary material for: Colour Doppler Ultrasonography in the Assessment of Intratesticular Lesions: Influence of Lesion Size and Vascular Pattern
Source: Cancers (Basel). 2026 Feb 25;18(5):741. doi: 10.3390/cancers18050741 (PMC12984502; doi:10.3390/cancers18050741)
Supplement: Supplementary file 1 [file cancers-18-00741-s001.zip › TableS1.pdf]

Table S1. Individual colour Doppler vascular pattern components in vascularised focal intratesticular lesions with Holm multiplicity control (n = 85).

Pattern component (present)	Group 1, n/N (%)	Group 2, n/N (%)	Unadjusted Fisher's p	Holm-adjusted p
**Neoplastic vs non-neoplastic (vascularised lesions only)**				
Peripheral vascular pattern	Neoplastic (n = 78): 9/78 (11.5)	Non-neoplastic (n = 7): 0/7 (0.0)	1.000	1.000
Criss-cross	Neoplastic (n = 78): 59/78 (75.6)	Non-neoplastic (n = 7): 3/7 (42.9)	0.082	0.246
Disordered/haphazard	Neoplastic (n = 78): 10/78 (12.8)	Non-neoplastic (n = 7): 0/7 (0.0)	0.592	1.000
**Benign vs malignant (vascularised lesions only)**				
Peripheral vascular pattern	Benign (n = 21): 3/21 (14.3)	Malignant (n = 64): 6/64 (9.4)	0.683	0.683
Criss-cross	Benign (n = 21): 13/21 (61.9)	Malignant (n = 64): 49/64 (76.6)	0.257	0.771
Disordered/haphazard	Benign (n = 21): 1/21 (4.8)	Malignant (n = 64): 9/64 (14.1)	0.439	0.878

Analysis is restricted to vascularised lesions (intralesional flow present; n = 85). Values are n/N (%) within each diagnostic group. Two-sided Fisher's exact tests are shown (unadjusted p). Holm correction was applied separately within each comparison family (neoplastic vs non-neoplastic; benign vs malignant) across the three individual pattern tests (peripheral, criss-cross, disordered/haphazard) to control the family-wise error rate. Pattern coding is presence/absence; peripheral vascularity was recorded independently (non-exclusive), while criss-cross and disordered/haphazard intralesional patterns were mutually exclusive and analysed as separate component tests.
