# Supplementary material for: Colour Doppler Ultrasonography in the Assessment of Intratesticular Lesions: Influence of Lesion Size and Vascular Pattern
Source: Cancers (Basel). 2026 Feb 25;18(5):741. doi: 10.3390/cancers18050741 (PMC12984502; doi:10.3390/cancers18050741)
Supplement: Supplementary file 1 [file cancers-18-00741-s001.zip › TableS2.pdf]

Table S2. Inter-observer agreement for colour Doppler vascular pattern assessment in vascularised focal intratesticular lesions (n = 85).

Vascular pattern	Cohen's κ	SE	Approximate 95% CI
**Peripheral vascularity**	0.587	0.140	0.31–0.86
**Criss-cross**	0.591	0.089	0.42–0.77
**Disordered/haphazard**	0.470	0.120	0.24–0.71
**Composite "disrupted" (criss-cross or disordered/haphazard)**	0.950	0.050	0.85–1.00

Agreement between the two readers was assessed using Cohen's κ (n = 85). Approximate 95% confidence intervals were calculated as κ ± 1.96 × SE (upper bounds capped at 1.00).
