# Supplementary material for: Colour Doppler Ultrasonography in the Assessment of Intratesticular Lesions: Influence of Lesion Size and Vascular Pattern
Source: Cancers (Basel). 2026 Feb 25;18(5):741. doi: 10.3390/cancers18050741 (PMC12984502; doi:10.3390/cancers18050741)
Supplement: Supplementary file 1 [file cancers-18-00741-s001.zip › TableS3.pdf]

Table S3. Seminoma versus Leydig cell tumour (vascularised subset): colour Doppler vascular pattern component comparisons with Holm multiplicity control.

Pattern	Seminoma (N = 34), n/N (%)	Leydig cell tumour (N = 14), n/N (%)	Unadjusted Fisher's p	Holm-adjusted p*
**Peripheral vascularity (present)**	4/34 (11.8)	3/14 (21.4)	0.400	0.400
**Criss-cross (present)**	33/34 (97.1)	10/14 (71.4)	0.021	0.063
**Disordered/haphazard (present)**	1/34 (2.9)	1/14 (7.1)	0.292	0.584
**Composite "disrupted" (criss-cross or disordered/haphazard)†**	34/34 (100.0)	11/14 (78.6)	0.021	-

Analysis is restricted to vascularised lesions (intralesional flow present) within the seminoma versus Leydig cell tumour subgroup (seminoma N = 34; Leydig cell tumour N = 14). Values are n/N (%) within each histology group. Two-sided Fisher's exact tests are shown (unadjusted p).

*Holm correction was applied within this subgroup comparison across the three individual component patterns (peripheral vascularity, criss-cross, disordered/haphazard).

† The composite "disrupted" endpoint was analysed as a separate clinical descriptor and was not included in the Holm family.
